# Human Respiratory Syncytial Virus in Vaccinated and Unvaccinated Adults, Georgia, USA, 2024–2025

**DOI:** 10.3201/eid3205.251997

**Published:** 2026-05

**Authors:** Saïd Rachida, Alaa Ahmed, Diana Rojas-Gallardo, Henok Tafesse, Hannah Dakanay, Mackenzie Duford, Collin Tolbert, Ryan S. Springfield, Anne Piantadosi

**Affiliations:** Emory University School of Medicine, Atlanta, Georgia, USA (S. Rachida, A. Ahmed, D. Rojas-Gallardo, H. Dakanay, M. Duford, C. Tolbert, R.S. Springfield, A. Piantadosi); Morehouse School of Medicine, Atlanta (H. Tafesse, C. Tolbert); Medical College of Georgia, Athens, Georgia, USA (R.S. Springfield)

**Keywords:** Respiratory syncytial virus, viruses, respiratory infections, genetic diversity, vaccine, phylogenetics, United States

## Abstract

We analyzed respiratory syncytial virus genome sequences from adults in Georgia, USA, during 2024–2025. We found multiple co-circulating lineages of both A and B subtypes. We identified few mutations in F protein antigenic sites in this population with low vaccine uptake, highlighting the need for ongoing genomic surveillance.

Respiratory syncytial virus (RSV) is a leading cause of respiratory tract infections in infants, older adults, and immunocompromised persons (*1*). Vaccines are available for adults on the basis of age and underlying conditions (*2*). In the United States during 2024–2025, 47.5% of adults >75 years of age and 38.1% of adults 60–74 years of age with high-risk conditions received RSV vaccinations (*3*). RSV vaccines target the fusion (F) protein at conserved epitopes; however, vaccination might create selective pressure for immune escape mutations.

Subtypes RSV-A and RSV-B have been classified into lineages (*4*), enabling surveillance for immune escape variants that might arise across diverse viral genetic backgrounds. US studies during 2022–2024 identified few substitutions in antigenic sites, none of which were clearly associated with vaccination (*5*–*7*). We analyzed RSV sequences during 2024–2025 to assess virus diversity under a changing immune landscape.

## The Study

Our study included 182 vaccinated and unvaccinated adults within the Emory Healthcare system, in Georgia, USA (Appendix 1); 68.7% were female, 30.7% were male, and the median age was 61 years (Appendix 1 Table 1). Nearly all (98%) persons reported symptoms, including fever (31%), cough (93%), and dyspnea (25%). Hospitalization occurred in 13%, intensive care unit admission in 2%, and death in 3% (Appendix 1 Table 3).

Ninety-six (53%) persons were eligible for RSV vaccination on the basis of age (≥75 years) or age 50–74 years with underlying conditions (*2*); however, only 17 (18% of eligible persons) received vaccinations. We did not perform statistical analyses because of the small sample size, but observed that vaccinated persons were older and had more underlying conditions, which likely contributed to their higher rates of hospitalization and intensive care admission (Appendix 1 Table 1). We detected similar numbers of RSV-A (n = 93) and RSV-B (n = 83) cases. We successfully sequenced 71% of RSV-A samples and 76% of RSV-B samples (Appendix 1 Table 2), generally corresponding to those with quantitative reverse transcription PCR cycle threshold values <31 (Appendix 1 Figure 1).

Among RSV-A sequences, lineage A.D.3.1 was predominant (n = 27; 29%), followed by A.D.5.2 (n = 14; 15%) and A.D.1.5 (n = 9; 10%) (Appendix 1 Table 4). Lineage A.D.3.1 was more frequent in our study than previously reported (*8*,*9*). Among RSV-B sequences, most were lineage B.D.E.1 (n = 48; 58%), consistent with other studies (*4*). Among 17 vaccinated persons, 14 had RSV sequences with >75% coverage; those sequences represented multiple lineages, with no clear differences in lineage distribution between vaccinated and unvaccinated persons (Appendix 1 Table 4). Phylogenetic analysis showed that sequences from our study were distributed across phylogenetic trees (Figure; Appendix 1 Figure 2), although we observed clusters of Georgia sequences (Appendix 1 Table 4). Sequences from vaccinated persons did not show distinct clustering.

**Figure:**
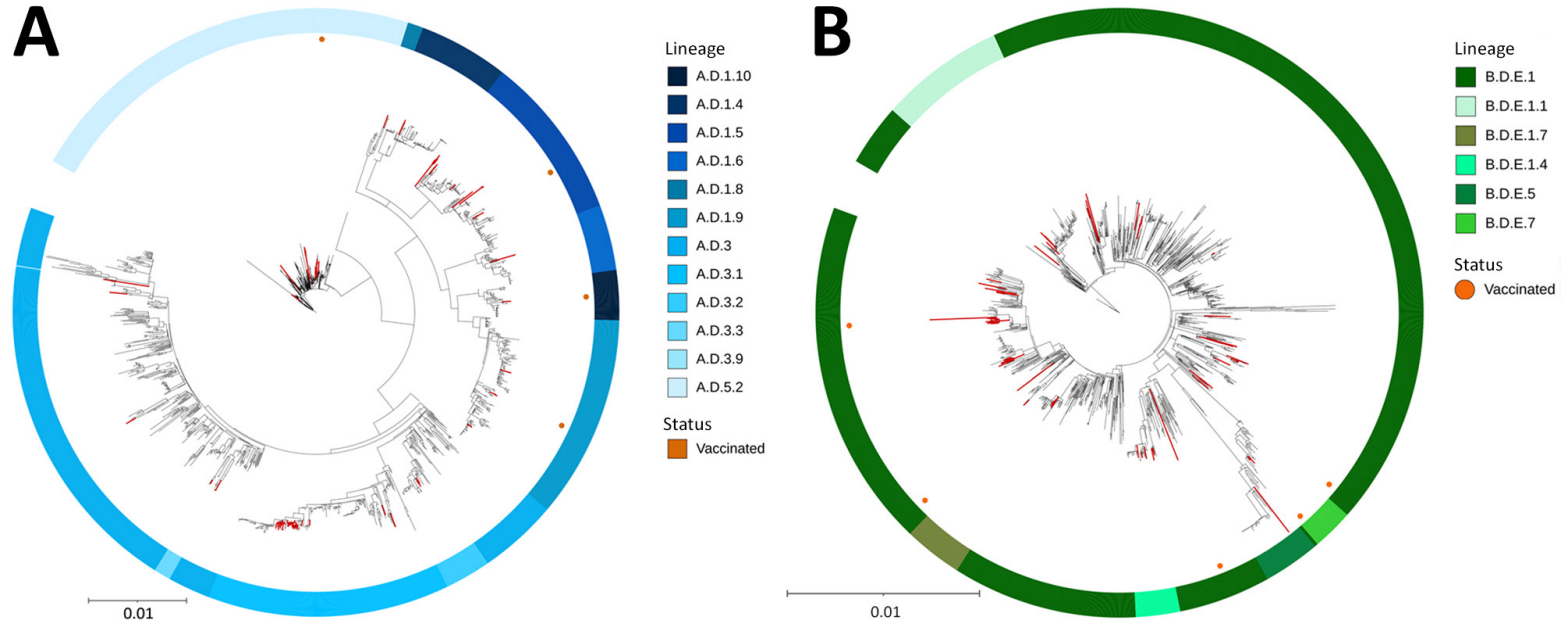
Maximum-likelihood phylogenetic trees of respiratory syncytial virus (RSV) A and RSV-B sequences from adults with RSV infection in Georgia, USA, 2024–2025. A) RSV-A sequences mainly clustered within lineages A.D.3.1 and A.D.5.2. B) RSV-B sequences mainly clustered within lineage B.D.E.1. There was limited clustering of the Georgia sequences obtained in this study (red branches), and RSV sequences from vaccinated persons (orange dots) were distributed throughout the tree. For ease of visualization, Panel B of this figure omits lineage B.D.4.1.1; that lineage is included in Appendix 1 Figure 2. Reference sequences are listed in Appendix 2. Scale bars indicate nucleotide substitutions per site.

We evaluated mutations in F protein antigenic sites Ø–V for 125 sequences with >95% F gene coverage, 13 of which came from vaccinated persons. Across all persons, we identified a total of 25 nonsynonymous substitutions in antigenic sites (Table; Appendix 1 Figures 3, 4). Only 1 substitution was unique to vaccinated persons, K65R in antigenic site Ø in 1 RSV-A sequence. K65R phenotypic effects are unknown, but other substitutions at this site have been associated with nirsevimab resistance (*10*). We found another substitution, S377N, in 43% of vaccinated and 5% of unvaccinated persons, a finding also noted in a report of 2 postvaccine infections during 2023–2024 (*6*), but phenotypic effects of that substitution are unknown. The other substitutions occurred at the same or higher frequency in unvaccinated compared with vaccinated persons.

**Table:** Amino acid mutations at antigenic sites in the RSV F protein for a study of human respiratory syncytial virus in vaccinated and unvaccinated adults, Georgia, USA, 2024–2025*

Antigenic site	RSV-A						RSV-B				
	Unvaccinated, n = 58			Vaccinated, n = 7			Unvaccinated, n = 53			Vaccinated, n = 6	
Sub.	No. (%)	Sub.	No. (%)		Sub.	No. (%)	Sub.	No. (%)
Site ϕ, 62–96, 195–227	T72I	1 (2)		K65R	1 (20)		N63S	1 (2)		I206M	6 (100)
							I206M	53 (100)		Q209R	5 (83)
							Q209R	52 (98)		S211N	6 (100)
							S211N	53 (100)			
Site I, 27–45, 312–318, 378–389	I379V	58 (100)		I379	7 (100)		R42K	25 (47)		R42K	2 (33)
	V384I	58 (100)		V384I	6 (80)		F54L	53 (100)		F54L	6 (100)
							S389P	50 (94)		S389P	4 (67)
Site II, 254–277	N276S	33 (57)		N276S	4 (57)		ND	ND		ND	ND
Site III, 46–54, 301–311, 345–352, 367–378	S377N	3 (5)		S377N	3 (43)		ND	ND		ND	ND
Site IV, 422–471	M447V	58 (100)		M447V	7 (100)		K445R	1 (2)		ND	ND
	K470R	2 (3)					N466S	1 (2)			
Site V, 55–61, 146–194, 287–300	I59V	5 (7)		V152I	7 (100)		L172Q	53 (100)		L172Q	6 (100)
	V152I	58 (100)		L178V	7 (100)		S173L	53 (100)		S173L	6 (100)
	V154I	1 (2)					S190N	52 (98)		S190N	6 (98)
	L178V	58 (100)					K191R	53 (100)		K191R	6 (100)

*Sub., substitution; RSV, respiratory syncytial virus.

Among all persons, several substitutions (e.g., I59V and K470R) in RSV-A occurred at higher frequencies than previously reported (*5*). For RSV-B, 9 of the 13 substitutions were characteristic of the B.D.E.1 lineage (*4*). R42K, detected in nearly half of our samples, was also frequently detected in prior reports (*6*); however, F54L, detected in all our sequences, was previously rarely reported. We identified 3 substitutions not reported in prior studies: N63S in site Ø, and K445R and N466S in site IV (*5**–**7*,*9*). Mutations at antigenic sites 384 of RSV-A and 191 of RSV-B, rarely noted in cases of postvaccine infection reported during 2023–2024 (*6*), were widely circulating in our cohort by 2024–2025. Although not likely to be a direct result of immune pressure, these findings highlight ongoing drift in the F protein that might have future consequences for vaccine effectiveness. We also analyzed within-sample minor variants in F and identified only 1 substitution in antigenic site G71E in 37% of reads from an unvaccinated person with RSV-A (Appendix 1 Table 5).

## Conclusions

Our analyses demonstrated that RSV strains circulating in Georgia during the 2024–25 season were diverse and resembled strains circulating across the United States and globally. We did not find evidence of vaccine-driven evolution; however, a primary limitation of this study was the small number of vaccinated persons. Continued large-scale RSV genomic surveillance will be critical for detecting emerging immune-escape variants and understanding viral evolution in the postvaccine era.

Appendix 1 Additional information for human respiratory syncytial virus in vaccinated and unvaccinated adults, Georgia, USA, 2024–2025.

Appendix 2 Full list of reference sequences for human respiratory syncytial virus in vaccinated and unvaccinated adults, Georgia, USA, 2024–2025.
