# Supplementary material for: Human Respiratory Syncytial Virus in Vaccinated and Unvaccinated Adults, Georgia, USA, 2024–2025
Source: Emerg Infect Dis. 2026 May;32(5):827–9. doi: 10.3201/eid3205.251997 (PMC13175093; doi:10.3201/eid3205.251997)
Supplement: Appendix 1 — Additional information for human respiratory syncytial virus in vaccinated and unvaccinated adults, Georgia, USA, 2024–2025. [file 25-1997-Techapp-s1.pdf]

Article DOI: http://doi.org/10.3201/eid3205.251997

EID cannot ensure accessibility for supplementary materials supplied by authors. Readers who have difficulty accessing supplementary content should contact the authors for assistance.

Human Respiratory Syncytial Virus in Vaccinated and Unvaccinated Adults, Georgia, USA, 2024–2025

Appendix

Methods

Study cohort

A randomly selected convenience set of 182 residual upper respiratory tract swabs positive for RSV by the Xpert CoV-2/Flu/RSV assay (Cepheid) were collected from patients tested in the Emory Healthcare system from September 2024 to March 2025, out of 1,140 total positive samples during this period. The number of samples collected per month were: September n=1 collected (out of 28 positives, 4%); October n=11 collected (out of 61 positives, 18%); November n=27 collected (out of 316 positives, 9%); December n=61 collected (out of 438 positives, 14%); January n=42 collected (out of 351 positives, n=12%), February n=30 collected (out of 157 collected, 19%); March n=10 collected (out of 109 positives, 9%) (Appendix 1 Table 2). Clinical and demographic data, including vaccine status, were obtained by manual review of electronic medical records. Comparisons were descriptive owing to the small number of vaccinated participants. This study was approved by the Emory Institutional Review Board (STUDY00007680).

RSV whole genome sequencing

Total nucleic acid was extracted from residual upper respiratory tract swabs using the Abbott m2000sp instrument and Abbott mSample Preparation Systems for RNA (Abbott, Abbott Park, IL, USA), following the manufacturer’s protocol. Extracted nucleic acid was treated with DNase using the Heat&Run® gDNA Removal Kit (ArcticZymes Technologies ASA, Norway). cDNA synthesis was performed with random hexamer primers and the SuperScript IV reverse transcriptase system (Invitrogen, Thermo Fisher Scientific, Waltham, MA, USA). Dual-indexed

multiplex amplicon libraries were generated with the xGen™ Respiratory Virus Amplicon Panel (Integrated DNA Technologies, Coralville, IA, USA) according to manufacturer's protocol, with the following modifications: multiplex PCR amplification was performed for 24 cycles followed by a 1X AMPure XP bead cleanup (Beckman Coulter, Brea, CA, USA). Sequencing adapters and unique dual indexes were added with an additional 8-cycle PCR using the xGen™ Amplicon UDI Primers, and libraries underwent a 0.65X AMPure XP bead cleanup. Final libraries were quantified with the KAPA Library Quantification Kit (Roche, Indianapolis, IN, USA), normalized to 4 nM, pooled in equimolar ratios, and subjected to a 0.8X AMPure XP bead cleanup. Negative controls (nuclease-free water samples) were processed alongside each batch. Sequencing was performed on Illumina MiSeq or NextSeq 2000 instruments.

RSV genome assembly

Sequencing reads were processed and underwent reference-based assembly using the nf-core/viralrecon pipeline (v2.5) (*1*), implemented in Nextflow (v22.10.5) (*2*). The workflow was configured for Illumina amplicon data using iVar (*3*) for primer trimming, variant calling, and consensus sequence generation. Primer sequences were removed using a 5-bp offset to reduce primer-derived artifacts, and bases with quality scores below 20 were filtered. Host-derived reads were removed using Kraken2 (*4*) against a human reference database. Remaining reads were aligned to subtype-specific reference genomes (RSV-A: GenBank OP890336.1 and PP681262.1; RSV-B: OP965707.1 and OZ280358.1) using Bowtie2 (*5*). Variants were called with iVar, and consensus sequences were generated for sites with ≥10X coverage and a minimum variant frequency of 0.75. Coverage metrics, including mean depth and percentage of genome covered at ≥1X and ≥10X, were compiled using MultiQC (*6*). Assemblies were considered successful when >75% of the reference genome was recovered at ≥10X coverage and the mean depth exceeded 100X.

Phylogenetic analyses

One hundred twenty-nine successfully assembled genomes were included in the phylogenetic analyses. For initial analyses, we used the reference datasets available in the Nextstrain (*7*) RSV builds for both subtypes A and B. Sequences were filtered using augur, retaining only those with ≥75% genome coverage, a "good" qc overallStatus, and belonging to the same clades identified in our samples. The full list of reference sequences is provided in Appendix 2 (https://wwwnc.cdc.gov/EID/article/32/5/25-1997-App2.xlsx).

Subsequently, genetic proximity–based subsampling was performed. Genetic distances between the reference sequences and the focal sequences from our study were calculated using the JC69 metric, and up to 40 sequences per country per year were retained. The resulting subsampled dataset was aligned using MAFFT (*8*), and maximum-likelihood phylogenetic trees were inferred with IQ-TREE (*9*), employing automatic selection of the best-fit substitution model (RSV-A: GTR+F+I+R4; RSV-B: GTR+F+I+R7) and 1000 ultrafast bootstrap replicates to assess node support.

Ultimately, the RSV-A dataset comprised 1,505 sequences, which included the lineages A.D.1.10, A.D.1.4, A.D.1.5, A.D.1.6, A.D.1.8, A.D.1.9, A.D.3, A.D.3.1, A.D.3.2, A.D.3.3, A.D.3.9, and A.D.5.2 (Figure 1, panel A [main text]). For RSV-B, two phylogenetic trees were generated: one using a dataset containing 2,053 sequences (Appendix 1 Figure 2), including the lineages B.D.4.1.1, B.D.E.1, B.D.E.5, B.D.E.7, B.D.E.1.1, B.D.E.1.4, and B.D.E.1.7; and another using a reduced dataset excluding the B.D.4.1.1 lineage, consisting of 1,034 sequences (Figure, panel B [main text]). The smaller RSV-B dataset was used to improve visualization, as the B.D.4.1.1 lineage was highly overrepresented in the full dataset. Final trees were annotated and visualized using iTOL (*10*). Rectangular trees are available at https://doi.org/10.5281/zenodo.18942899.

F gene substitution analyses

Sequences with ≥95% coverage of the F gene from 98 individuals were aligned using MAFFT v7 (*8*) and visually inspected in BioEdit v7 (*11*) to identify consensus-level substitutions. Minor variants within the F gene were identified by iVar and filtered to meet the following thresholds: alternative allele frequency (ALT_FREQ) ≥ 5%, total depth (TOTAL_DP) ≥ 100, and base quality scores ≥ 30 for both reference and alternative alleles. Additionally, each variant had to be supported by at least 20 alternative reads, with ≥10 reads on each strand, and pass both Fisher's exact test for sequencing error (PVAL ≥ 0.05) and the Fisher strand-bias test (PVAL ≥ 0.05). A synonymous C→T substitution at nucleotide position 5799, corresponding to amino acid position 37 of the F gene was detected in six RSV-A genomes. This variant passed all quality thresholds, however further inspection of the BAM files revealed that the supporting reads frequently mapped near the ends of amplicons/read boundaries. Due to this positional bias and the likelihood of mispriming or end-of-read errors, we did not consider this variant reliable and therefore excluded it from the final reported variant table.

References

1. Ewels PA, Peltzer A, Fillinger S, Patel H, Alneberg J, Wilm A, et al. The nf-core framework for community-curated bioinformatics pipelines. Nat Biotechnol. 2020;38:276–8. PubMed https://doi.org/10.1038/s41587-020-0439-x

2. Di Tommaso P, Chatzou M, Floden EW, Barja PP, Palumbo E, Notredame C. Nextflow enables reproducible computational workflows. Nat Biotechnol. 2017;35:316–9. PubMed https://doi.org/10.1038/nbt.3820

3. Grubaugh ND, Gangavarapu K, Quick J, Matteson NL, De Jesus JG, Main BJ, et al. An amplicon-based sequencing framework for accurately measuring intrahost virus diversity using PrimalSeq and iVar. Genome Biol. 2019;20:8. PubMed https://doi.org/10.1186/s13059-018-1618-7

4. Wood DE, Lu J, Langmead B. Improved metagenomic analysis with Kraken 2. Genome Biol. 2019;20:257. PubMed https://doi.org/10.1186/s13059-019-1891-0

5. Langmead B, Salzberg SL. Fast gapped-read alignment with Bowtie 2. Nat Methods. 2012;9:357–9. PubMed https://doi.org/10.1038/nmeth.1923

6. Ewels P, Magnusson M, Lundin S, Käller M. MultiQC: summarize analysis results for multiple tools and samples in a single report. Bioinformatics. 2016;32:3047–8. PubMed https://doi.org/10.1093/bioinformatics/btw354

7. Hadfield J, Megill C, Bell SM, Huddleston J, Potter B, Callender C, et al. Nextstrain: real-time tracking of pathogen evolution. Bioinformatics. 2018;34:4121–3. PubMed https://doi.org/10.1093/bioinformatics/bty407

8. Katoh K, Standley DM. MAFFT multiple sequence alignment software version 7: improvements in performance and usability. Mol Biol Evol. 2013;30:772–80. PubMed https://doi.org/10.1093/molbev/mst010

9. Nguyen LT, Schmidt HA, von Haeseler A, Minh BQ. IQ-TREE: a fast and effective stochastic algorithm for estimating maximum-likelihood phylogenies. Mol Biol Evol. 2015;32:268–74. PubMed https://doi.org/10.1093/molbev/msu300

10. Letunic I, Bork P. Interactive Tree of Life (iTOL) v6: recent updates to the phylogenetic tree display and annotation tool. Nucleic Acids Res. 2024;52(W1):W78–82. PubMed https://doi.org/10.1093/nar/gkae268

11. Hall T, Biosciences I, Carlsbad CJ. BioEdit: an important software for molecular biology. GERF Bull Biosci. 2011;2:60–1.

Appendix 1 Table 1. Summary of demographic and clinical characteristics of vaccinated and unvaccinated adults with human respiratory syncytial virus infection, Emory Healthcare system, 2024–2025 season.

Characteristic	Total (N = 182)	Unvaccinated (N = 165)	Vaccinated (N = 17)
RSV subtype			
RSV-A	93	85	8
RSV-B	83	74	9
Not determined	6	6	0
Age, median (range)	61 (18–99)	59 (18–92)	73 (61–99)
Sex			
Female, no. (%)	125 (69)	112 (68)	13 (76)
Male, no. (%)	56 (31)	52 (32)	4 (24)
Unknown	1	1	0
Symptoms			
Any* (%)	178 (98)	157 (95)	17 (100)
Fever (%)	56 (31)	50 (30)	5 (29)
Cough (%)	169 (93)	150 (91)	15 (88)
Dyspnea (%)	46 (25)	39 (24)	6 (35)
Comorbidities, no. (%)	119 (65)	102 (62)	14 (82)
Immunocompromised, no. (%)	16 (9)	15 (9)	1 (6)
Outcomes			
Hospitalized (%)	24 (13)	21 (13)	3 (18)
ICU (%)	4 (2)	3 (2)	1 (6)
Death (%)	6 (3)	6 (4)	0 (0)

ICU= intensive care unit.

Appendix 1 Table 2. Sequencing metrics for samples in this study

Sample ID	Collection date	Ct value	Total reads	Genome coverage >10x (%)	Average depth	NCBI accession no	Lineage
PGCoE_0421E	2024-09-21	35.5	392,196	1	NA	NA	
PGCoE_0431O	2024-10-02	30.3	2,058,754	69	51	NA	
PGCoE_0436T	2024-10-04	22.1	15,102,814	99	14,975	PX639978	B.D.E.1
PGCoE_0440X	2024-10-07	24.9	3,464,904	97	4,124	PX639943	A.D.1.9
PGCoE_0447E	2024-10-16	23.8	20,687,008	100	28,776	PX639979	B.D.E.1
PGCoE_0455M	2024-10-18	28.4	466,618	90	154	PX639980	B.D.E.1
PGCoE_0462T	2024-10-23	33.6	442,712	33	4	NA	
PGCoE_0470B	2024-10-27	29.2	605,472	8	NA	NA	
PGCoE_0471C	2024-10-28	31.9	342,816	59	18	NA	
PGCoE_0472D	2024-10-28	24.6	1,738,846	97	1,442	PX639995	B.D.E.1
PGCoE_0474F	2024-10-28	24.5	7,145,594	99	8,825	PX639977	B.D.E.1
PGCoE_0475G	2024-10-28	30.6	1,106,720	95	349	PX639996	B.D.E.1
PGCoE_0484P	2024-11-02	32.8	556,596	9	NA	NA	
PGCoE_0486R	2024-11-05	35.8	531,358	12	NA	NA	
PGCoE_0487S	2024-11-05	22.1	3,045,270	98	1,977	PX639997	B.D.E.1
PGCoE_0488T	2024-11-06	17.4	12,046,354	99	8,044	PX639998	B.D.4.1.1
PGCoE_0490V	2024-11-06	18.9	14,414,334	99	10,785	PX639999	B.D.E.1
PGCoE_0491W	2024-11-07	22.2	15,357,648	100	14,521	PX639932	A.D.5.2
PGCoE_0492X	2024-11-07	23.7	15,276,212	99	6,259	PX640000	B.D.E.1
PGCoE_0495A	2024-11-08	21.7	13,728,548	98	11,734	PX639933	A.D.1.5
PGCoE_0496B	2024-11-08	27.7	2,130,638	88	346	PX639934	A.D.3.9
PGCoE_0497C	2024-11-08	31.1	2,262,454	60	16	NA	
PGCoE_0499E	2024-11-11	20.4	17,416,196	98	19,759	PX639965	A.D.3.1
PGCoE_0500F	2024-11-11	33.4	1,335,532	75	39	PX640001	B.D.E.1
PGCoE_0502H	2024-11-11	17.4	18,776,370	100	23,185	PX640002	B.D.E.1
PGCoE_0505K	2024-11-12	28.4	944,038	88	216	PX639909	A.D.3.1
PGCoE_0507M	2024-11-12	34.9	399,918	23	3	NA	
PGCoE_0508N	2024-11-13	20.2	18,526,330	99	13,816	PX640003	B.D.E.1
PGCoE_0511Q	2024-11-13	33.3	2,503,098	28	4	NA	
PGCoE_0513S	2024-11-13	18.2	11,171,542	98	8,830	PX639910	A.D.1.5
PGCoE_0515U	2024-11-15	22.3	3,634,742	90	184	PX639981	B.D.E.1
PGCoE_0520Z	2024-11-19	20.8	2,770,616	95	374	PX639985	B.D.E.1
PGCoE_0521A	2024-11-19	22.7	2,562,860	94	526	PX639986	B.D.E.1
PGCoE_0523C	2024-11-20	24.5	1,621,506	64	49	NA	
PGCoE_0525E	2024-11-20	24.6	1,878,352	47	8	NA	
PGCoE_0526F	2024-11-21	22.8	3,995,988	93	328	PX639982	B.D.E.1.7
PGCoE_0527G	2024-11-21	25	1,995,948	97	1,340	PX639987	B.D.4.1.1
PGCoE_0533M	2024-11-26	25.5	2,561,210	95	1,152	PX639988	B.D.E.1
PGCoE_0537Q	2024-11-29	23.4	2,499,096	98	1,889	PX639927	A.D.5.2

Sample ID	Collection date	Ct value	Total reads	Genome coverage >10x (%)	Average depth	NCBI accession no	Lineage
PGCoE_0538R	2024-12-02	36	297,502	52	12	NA	
PGCoE_0541U	2024-12-02	18.6	2,728,912	98	1,332	PX639989	B.D.E.1
PGCoE_0542V	2024-12-02	30.4	172,496	78	70	PX639901	A.D.3.1
PGCoE_0543W	2024-12-02	16.7	3,123,724	96	1,820	PX639902	A.D.3.1
PGCoE_0544X	2024-12-03	21.3	2,620,722	96	2,259	PX639928	A.D.3
PGCoE_0546Z	2024-12-03	26.3	496,746	94	389	PX639929	A.D.5.2
PGCoE_0547A	2024-12-04	24.2	2,547,998	97	3,165	PX639903	A.D.3.1
PGCoE_0550D	2024-12-04	31.8	132,812	49	9	NA	
PGCoE_0551E	2024-12-04	NR	176,552	73	31	NA	
PGCoE_0552F	2024-12-05	20.1	2,498,180	97	778	PX640004	B.D.E.1.1
PGCoE_0554H	2024-12-05	34.4	158,352	NA	NA	NA	
PGCoE_0561O	2024-12-06	25.7	374,026	78	55	PX640005	B.D.E.1
PGCoE_0564R	2024-12-07	31.6	668,964	85	91	PX639935	A.D.5.2
PGCoE_0566T	2024-12-08	22.9	2,824,978	89	191	PX639967	B.D.E.1
PGCoE_0567U	2024-12-09	19.6	5,393,656	95	465	PX639968	B.D.E.1
PGCoE_0571Y	2024-12-09	19.2	3,082,544	94	1,355	PX639911	A.D.1.9
PGCoE_0572Z	2024-12-09	28.4	2,108,116	87	288	PX639946	A.D.3.1
PGCoE_0575C	2024-12-11	31.4	564,594	77	80	PX639936	A.D.5.2
PGCoE_0576D	2024-12-11	35	570,842	14	NA	NA	
PGCoE_0577E	2024-12-11	31.1	472,510	45	8	NA	
PGCoE_0579G	2024-12-11	26.8	2,905,824	95	1,973	PX639947	A.D.3.1
PGCoE_0580H	2024-12-11	35.3	405,268	24	2	NA	
PGCoE_0581I	2024-12-11	33.1	260,294	NA	NA	NA	
PGCoE_0583K	2024-12-12	22.8	2,426,554	97	1,055	PX640006	B.D.E.1
PGCoE_0584L	2024-12-12	24.1	2,309,774	97	1,308	PX639937	A.D.5.2
PGCoE_0585M	2024-12-12	33.7	590,640	17	1	NA	
PGCoE_0586N	2024-12-12	27.5	3,449,050	99	1,938	PX640021	B.D.E.1
PGCoE_0589Q	2024-12-12	34.6	645,274	3	NA	NA	
PGCoE_0593U	2024-12-12	NR	468,590	11	NA	NA	
PGCoE_0594V	2024-12-13	25.1	771,632	89	193	PX640007	B.D.E.1
PGCoE_0595W	2024-12-13	28.8	536,434	76	36	PX639969	B.D.E.1
PGCoE_0596X	2024-12-13	34.8	497,432	33	4	NA	
PGCoE_0597Y	2024-12-13	31.3	422,790	10	NA	NA	
PGCoE_0599A	2024-12-13	22.1	1,707,976	93	900	PX639938	A.D.1.6
PGCoE_0603E	2024-12-13	22.7	1,637,210	93	761	PX639912	A.D.3.1
PGCoE_0606H	2024-12-14	17.8	1,136,582	91	397	PX639913	A.D.3.1
PGCoE_0607I	2024-12-14	18.2	2,352,218	97	887	PX640008	B.D.E.1
PGCoE_0611M	2024-12-15	21.2	1,692,920	91	507	PX639914	A.D.3.1
PGCoE_0617S	2024-12-16	26.2	534,846	89	169	PX640009	B.D.E.1
PGCoE_0619U	2024-12-16	31.2	565,008	68	21	NA	
PGCoE_0622X	2024-12-16	18.2	2,487,408	96	764	PX640010	B.D.E.1
PGCoE_0623Y	2024-12-16	18.5	2,509,648	95	857	PX640011	B.D.E.1.4
PGCoE_0626B	2024-12-16	37.7	358,952	15	NA	NA	
PGCoE_0627C	2024-12-16	20.6	1,990,654	92	960	PX639915	A.D.3.1
PGCoE_0629E	2024-12-16	22.6	705,230	80	118	PX639916	A.D.3.1
PGCoE_0631G	2024-12-17	25	1,673,246	95	871	PX639939	A.D.5.2
PGCoE_0635K	2024-12-18	30.9	488,668	68	30	NA	
PGCoE_0636L	2024-12-18	35.8	406,858	1	NA	NA	
PGCoE_0639O	2024-12-18	21.6	1,607,908	91	624	PX639917	A.D.1.5
PGCoE_0647W	2024-12-19	25.2	644,642	88	118	PX640012	B.D.E.1
PGCoE_0648X	2024-12-19	30.8	862,936	92	333	PX639940	A.D.5.2
PGCoE_0659I	2024-12-19	20.2	1,062,516	91	330	PX640013	B.D.E.1
PGCoE_0661K	2024-12-19	25	11,009,062	98	8,918	PX639948	A.D.3.1
PGCoE_0666P	2024-12-30	35	477,292	11	NA	NA	
PGCoE_0669S	2024-12-30	20.3	2,301,094	95	492	PX640014	B.D.E.1.7
PGCoE_0670T	2024-12-30	23.3	826,354	80	196	PX639941	A.D.1.9
PGCoE_0674X	2024-12-30	17.6	1,875,676	83	200	PX639918	A.D.1.9
PGCoE_0683G	2024-12-30	18.9	6,112,918	94	352	PX639970	B.D.E.7
PGCoE_0691O	2024-12-31	35.2	592,016	NA	NA	NA	
PGCoE_0693Q	2024-12-31	20	1,325,780	75	65	PX639966	A.D.1.5
PGCoE_0699W	2024-12-31	24.8	610,644	9	NA	NA	
PGCoE_0720R	2025-01-02	21.5	6,504,408	96	2,344	PX639919	A.D.3.1
PGCoE_0722T	2025-01-02	33.1	1,083,482	27	2	NA	
PGCoE_0726X	2025-01-03	25.6	2,420,884	87	369	PX639920	A.D.3.1
PGCoE_0728Z	2025-01-03	34.3	1,724,744	39	4	NA	
PGCoE_0731C	2025-01-03	19.3	11,108,304	99	1,632	PX640015	B.D.E.1.7
PGCoE_0735G	2025-01-03	23.1	2,620,190	91	306	PX639971	B.D.E.1

Sample ID	Collection date	Ct value	Total reads	Genome coverage >10x (%)	Average depth	NCBI accession no	Lineage
PGCoE_0739K	2025-01-03	28.3	11,323,936	100	9,080	PX639949	A.D.5.2
PGCoE_0752X	2025-01-05	27.5	4,493,614	94	941	PX640016	B.D.E.1
PGCoE_0768N	2025-01-06	33.3	1,895,416	47	8	NA	
PGCoE_0781A	2025-01-07	23.6	8,735,304	99	3,133	PX640017	B.D.E.1.7
PGCoE_0782B	2025-01-07	23.1	5,286,388	95	1,688	PX639921	A.D.3.1
PGCoE_0786F	2025-01-07	22.4	4,003,008	92	1,248	PX639922	A.D.1.5
PGCoE_0788H	2025-01-07	26.3	3,568,256	96	1,502	PX640022	B.D.E.1
PGCoE_0791K	2025-01-07	26.1	12,565,460	99	12,293	PX640023	B.D.E.1
PGCoE_0792L	2025-01-07	35.9	2,815,002	31	2	NA	
PGCoE_0793M	2025-01-07	21.1	9,460,128	95	3,496	PX639923	A.D.1.5
PGCoE_0794N	2025-01-07	27.3	3,250,142	95	975	PX639924	A.D.3.1
PGCoE_0804X	2025-01-08	26.1	2,820,858	94	258	PX640018	B.D.E.1.1
PGCoE_0821O	2025-01-08	26.8	3,660,958	95	859	PX639942	A.D.5.2
PGCoE_0843K	2025-01-09	27.6	1,864,432	94	604	PX639950	A.D.3.1
PGCoE_0851S	2025-01-09	36.5	5,144,416	NA	NA	NA	
PGCoE_0852T	2025-01-09	33.7	2,592,992	13	NA	NA	
PGCoE_0853U	2025-01-09	19.2	7,385,804	94	1,818	PX639925	A.D.1.10
PGCoE_0857Y	2025-01-12	28.7	2,968,204	NA	NA	NA	
PGCoE_0875Q	2025-01-13	34.4	1,977,310	29	2	NA	
PGCoE_0879U	2025-01-14	19.9	4,859,536	93	543	PX639972	B.D.E.1
PGCoE_0882X	2025-01-14	29.7	1,106,744	20	NA	NA	
PGCoE_0883Y	2025-01-14	33.9	1,603,112	3	NA	NA	
PGCoE_0885A	2025-01-14	16.7	3,636,804	92	900	PX639926	A.D.3.1
PGCoE_0899O	2025-01-14	21.8	3,103,608	91	226	PX639973	B.D.E.1
PGCoE_0903S	2025-01-15	37.7	2,153,316	1	NA	NA	
PGCoE_0905U	2025-01-15	25.9	3,447,676	87	142	PX640019	B.D.E.1.1
PGCoE_0912B	2025-01-16	20.6	6,460,950	95	1,131	PX639974	B.D.E.1
PGCoE_0922L	2025-01-16	23.2	3,752,550	92	344	PX640020	B.D.E.1
PGCoE_1032R	2025-01-26	21.6	2,155,498	93	1,329	PX639904	A.D.3.1
PGCoE_1047G	2025-01-27	20.4	2,395,776	94	1,820	PX639905	A.D.3.1
PGCoE_1070D	2025-01-28	25.9	1,719,858	88	544	PX639944	A.D.3.2
PGCoE_1095C	2025-01-29	22.2	9,295,174	97	7,912	PX639951	A.D.3.1
PGCoE_1114V	2025-01-29	21.5	3,804,640	96	1,048	PX639990	B.D.E.1
PGCoE_1140V	2025-01-30	30	571,458	58	14	NA	
PGCoE_1144Z	2025-01-30	30.9	576,542	44	7	NA	
PGCoE_1173C	2025-01-31	25.7	1,071,098	84	132	PX639991	B.D.E.1
PGCoE_1192V	2025-02-03	28.6	558,622	75	39	PX639992	B.D.E.1
PGCoE_1200D	2025-02-03	29.7	724,480	2	NA	NA	
PGCoE_1206J	2025-02-03	27.7	1,179,942	85	260	PX639952	A.D.1.5
PGCoE_1230H	2025-02-04	24.2	706,264	85	269	PX639906	A.D.3.1
PGCoE_1320T	2025-02-08	24.4	1,669,324	95	578	PX639993	B.D.E.1
PGCoE_1330D	2025-02-11	20.2	2,342,600	95	2,023	PX639907	A.D.3.1
PGCoE_1331E	2025-02-11	19.9	2,065,798	91	634	PX639930	A.D.5.2
PGCoE_1332F	2025-02-11	18.4	15,075,280	99	12,574	PX639953	A.D.3.3
PGCoE_1333G	2025-02-11	22.1	1,110,704	82	122	PX639908	A.D.1.5
PGCoE_1334H	2025-02-12	19.3	3,209,280	95	1,849	PX639931	A.D.3
PGCoE_1335I	2025-02-12	25.2	692,114	90	189	PX639994	B.D.E.1
PGCoE_1336J	2025-02-12	25.6	2,990,252	89	746	PX639945	A.D.1.5
PGCoE_1337K	2025-02-13	28.4	2,102,140	56	19	NA	
PGCoE_1391M	2025-02-14	27.2	2,563,552	91	440	PX639983	B.D.E.1
PGCoE_1395Q	2025-02-14	24.2	16,329,974	100	15,394	PX639954	A.D.5.2
PGCoE_1405A	2025-02-16	25	13,999,858	99	8,968	PX639955	A.D.5.2
PGCoE_1408D	2025-02-17	20.4	15,902,702	99	11,301	PX639956	A.D.3.1
PGCoE_1412H	2025-02-17	29.2	617,466	4	NA	NA	
PGCoE_1413I	2025-02-17	29.2	759,866	9	NA	NA	
PGCoE_1417M	2025-02-17	31.3	692,494	NA	NA	NA	
PGCoE_1439I	2025-02-20	25.7	2,014,008	95	849	PX640024	B.D.E.7
PGCoE_1442L	2025-02-20	25	3,508,658	98	1,650	PX640025	B.D.E.5
PGCoE_1445O	2025-02-22	21.4	11,428,800	98	7,702	PX639957	A.D.1.4
PGCoE_1451U	2025-02-24	26.2	1,766,916	95	1,075	PX639958	A.D.3.3
PGCoE_1462F	2025-02-24	30.1	2,078,362	3	NA	NA	
PGCoE_1469M	2025-02-25	24.9	2,515,160	97	1,518	PX639959	A.D.3.2
PGCoE_1473Q	2025-02-26	30.7	1,785,416	4	NA	NA	
PGCoE_1478V	2025-02-26	27.1	2,002,662	95	777	PX640026	B.D.E.1.4
PGCoE_1485C	2025-02-27	23.5	5,023,958	98	4,511	PX639960	A.D.3.3
PGCoE_1492J	2025-02-28	24.3	14,209,992	100	25,453	PX639961	A.D.5.2
PGCoE_1502T	2025-03-03	20.1	20,878,290	77	73	PX639975	B.D.E.1

Sample ID	Collection date	Ct value	Total reads	Genome coverage >10x (%)	Average depth	NCBI accession no	Lineage
PGCoE_1510B	2025-03-04	21.8	11,238,546	99	19,458	PX639962	A.D.3.1
PGCoE_1514F	2025-03-04	20.6	11,952,100	99	15,629	PX639963	A.D.1.8
PGCoE_1516H	2025-03-04	17.4	11,692,596	100	7,271	PX640027	B.D.E.1
PGCoE_1517I	2025-03-04	25.1	7,298,496	99	9,941	PX640028	B.D.E.1.4
PGCoE_1540F	2025-03-07	28	1,767,846	95	874	PX640029	B.D.E.1
PGCoE_1560Z	2025-03-11	17.7	22,335,912	75	56	PX639976	B.D.E.1
PGCoE_1563C	2025-03-11	22.1	12,665,826	98	9,359	PX639964	A.D.3.1
PGCoE_1574N	2025-03-13	29.8	10,966,676	17	NA	NA	
PGCoE_1673I	2025-03-26	27.4	1,798,832	89	241	PX639984	B.D.E.1

NR= not reported. NA= not applicable.

Appendix 1 Table 3. Demographic and clinical characteristics of individuals in this study, by RSV subtype.

Variable	Category	Total (N=182*)	RSV-A (N=93)	RSV-B (N=83)
Median age (IQR†)		61 (43-72.8)	61 (43-72)	63 (45.5-73)
Gender (%)	Female	125 (68.7%)	65 (69.9%)	58 (69.9%)
	Male	56 (30.8%)	27 (29%)	25 (30.1%)
	Unknown	1 (0.5%)	1 (1.1%)	0 (0%)
Race (%)	Asian	12 (6.7%)	6 (6.6%)	5 (6%)
	Black or African American	72 (40.0%)	37 (40.7%)	33 (39.8%)
	Other	6 (3.3%)	3 (3.3%)	3 (3.6%)
	Unknown	7 (3.9%)	4 (4.4%)	3 (3.6%)
	White	83 (46.1%)	41 (45.1%)	39 (47%)
Ethnicity (%)	Non-Hispanic/Latino	162 (89%)	76 (91.6%)	6 (100%)
	Hispanic/Latino	2(1.1%)	1 (1.2%)	0 (0%)
	Other/Multiple	2(1.1%	2 (2.4%)	0 (0%)
	Unknown	14(7.7%)	4 (4.8%)	0 (0%)
	Declined	2(1.1%)	0 (0%)	0 (0%)
Symptoms (%)	No	4 (2.2%)	2 (2.2%)	2 (2.4%)
	Yes	178 (97.8%)	91 (97.8%)	81 (97.6%)
Fever (%)	No	126 (69.2%)	58 (62.4%)	63 (75.9%)
	Yes	56 (30.8%)	35 (37.6%)	20 (24.1%)
Cough (%)	No	12 (6.6%)	5 (5.4%)	7 (8.4%)
	Yes	169 (93.4%)	87 (94.6%)	76 (91.6%)
Sputum (%)	No	118 (64.8%)	60 (64.5%)	56 (67.5%)
	Yes	64 (35.2%)	33 (35.5%)	27 (32.5%)
Shortness of breath (%)	No	136 (74.7%)	69 (74.2%)	63 (75.9%)
	Yes	46 (25.3%)	24 (25.8%)	20 (24.1%)
Sore throat (%)	No	111 (61%)	56 (60.2%)	51 (61.4%)
	Yes	71(39%)	37 (39.8%)	32 (38.6%)
Congestion/ Runny nose (%)	No	49 (26.9%)	27 (29%)	20 (24.1%)
	Yes	133 (73.1%)	66 (71%)	63 (75.9%)
Voice change (%)	No	176 (96.7%)	89 (95.7%)	81 (97.6%)
	Yes	6 (3.3%)	4 (4.3%)	2 (2.4%)
Wheezing (%)	No	153 (84.1%)	78 (83.9%)	70 (84.3%)
	Yes	29 (15.9%)	15 (16.1%)	13 (15.7%)
Ear pain (%)	No	161 (88.5%)	82 (88.2%)	74 (89.2%)
	Yes	21 (11.5%)	11 (11.8%)	9 (10.8%)
Nausea (%)	No	163 (89.6%)	84 (90.3%)	74 (89.2%)
	Yes	19 (10.4%)	9 (9.7%)	9 (10.8%)
Vomiting (%)	No	170 (93.4%)	86 (92.5%)	79 (95.2%)
	Yes	12 (6.6%)	7 (7.5%)	4 (4.8%)
Diarrhea (%)	No	178 (97.8%)	90 (96.8%)	82 (98.8%)
	Yes	4 (2.2%)	3 (3.2%)	1 (1.2%)
Headache (%)	No	147 (80.8%)	74 (79.6%)	67 (80.7%)
	Yes	35 (19.2%)	19 (20.4%)	16 (19.3%)
Fatigue (%)	No	134 (73.6%)	69 (74.2%)	61 (73.5%)
	Yes	48 (26.4%)	24 (25.8%)	22 (26.5%)
RSV vaccination (%)	No	165 (90.7%)	85 (91.4%)	74 (89.2%)
	Yes	17 (9.3%)	8 (8.6%)	9 (10.8%)
Co-morbidities (%)	No	65 (35.7%)	37 (39.8%)	27 (32.5%)
	Yes	117 (64.3%)	56 (60.2%)	56 (67.5%)
Immunocompromising condition (%)	No	166 (91.2%)	86 (92.5%)	76 (91.6%)
	Yes	16(8.8%)	7 (7.5%)	7 (8.4%)
Hospitalization (%)	No	158 (86.8%)	77 (82.8%)	76 (91.6%)
	Yes	24 (13.2%)	16 (17.2%)	7 (8.4%)
ICU (%)	No	178 (97.8%)	92 (98.9%)	80 (96.4%)
	Yes	4 (2.2%)	1 (1.1%)	3 (3.6%)
Mechanical ventilation (%)	No	180 (98.9%)	92 (98.9%)	82 (98.8%)
	Yes	2 (1.1%)	1 (1.1%)	1 (1.2%)
Death‡ (%)	No	176 (96.7%)	90 (96.8%)	81 (97.6%)
	Yes	6 (3.3%)	3 (3.2%)	2 (2.4%)

IQR= interquartile range.
*Includes six individuals with RSV subtype not able to be determined.
‡Death from RSV or its complication was determined by manual chart review.

Appendix 1 Table 4. Distribution of RSV lineages among vaccinated and unvaccinated adults with RSV infection in the Emory Healthcare system, 2024 - 2025 season

RSV subtype	Lineage	Total (N=129)	Unvaccinated (N=115)	Vaccinated (N=14)	Phylogenetic clusters of GA sequences
RSV-A	A.D.3.1	27	24	3	2 clusters of 10-12 samples each
	A.D.5.2	14	13	1	2 clusters of 4 samples each
	A.D.1.5	9	8	1	2 clusters of 2-3 samples each
	A.D.1.9	4	3	1	1 cluster of 2 samples
	A.D.3.3	3	3	0	1 cluster of 2 samples
	A.D.3	2	2	0	No clusters
	A.D.3.2	2	2	0	1 cluster of 2 samples
	A.D.1.10	1	0	1	N/A
	A.D.3.9	1	1	0	N/A
	A.D.1.6	1	1	0	N/A
	A.D.1.4	1	1	0	N/A
	A.D.1.8	1	1	0	N/A
RSV-B	B.D.E.1	48	44	4	8 clusters of 2-10 samples each
	B.D.E.1.7	4	4	0	1 cluster of 4 samples
	B.D.E.1.1	3	3	0	1 cluster of 2 samples
	B.D.E.1.4	3	2	1	No clusters
	B.D.E.7	2	1	1	1 cluster of 2 samples
	B.D.4.1.1	2	1	1	No clusters
	B.D.E.5	1	1	0	N/A

Appendix 1 Table 5. Summary of minor variants detected in RSV-A and RSV-B samples from individuals in this study.

Sample ID	Nucleotide position*	Gene	Nucleotide Change	Frequency†	Amino Acid Position‡	Reference Amino Acid‡	Mutant Amino Acid‡	Resulting Change‡	Potential Impact
RSV-A									
PGCoE_0603E	6903	F	C>T	0.39	405	Serine (S)	Serine (S)	--	--
PGCoE_0670T	5900	F	G>A	0.37	71	Glycine (G)	Glutamic Acid (E)	G71E	Altered fusogenicity, antibody escape (site Ø)
RSV-B									
PGCoE_0521A	5703	F	A>C	0.74	13	Leucine (L)	Leucine (L)	--	--
PGCoE_0521A	5973	F	T>C	0.92	103	Alanine (A)	Alanine (A)	--	--
PGCoE_1114V	6090	F	A>G	0.90	142	Leucine (L)	Leucine (L)	--	--
PGCoE_0583K	6933	F	T>A	0.61	423	Threonine (T)	Threonine (T)	--	--

*Nucleotide position relative to PP681262 (RSV-A) and OZ280358 (RSV-B).
†Frequency = proportion of sequencing reads carrying the variant.
‡Amino acid position relative to NC038235 (RSV-A) and NC001781 (RSV-B) - F protein.

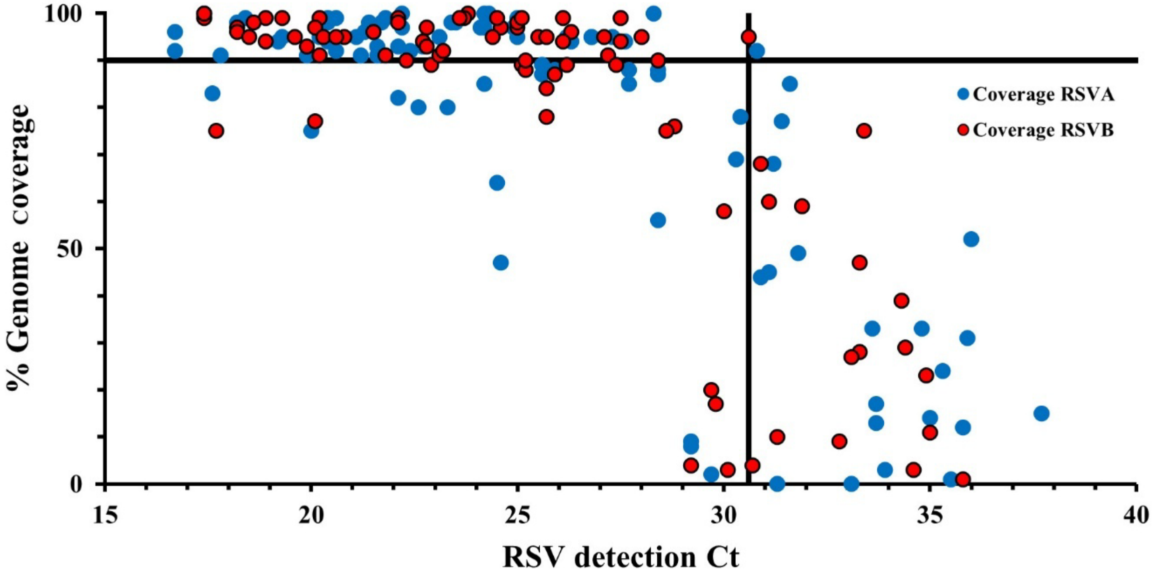

Appendix 1 Figure 1. Percent genome coverage (at 10X depth) vs. reverse-transcription quantitative PCR cycle threshold (Ct) values for the samples sequenced in this study. The horizontal line represents 90% genome coverage, and the vertical line represents a Ct value of 30.8. RSV, Respiratory Syncytial Virus

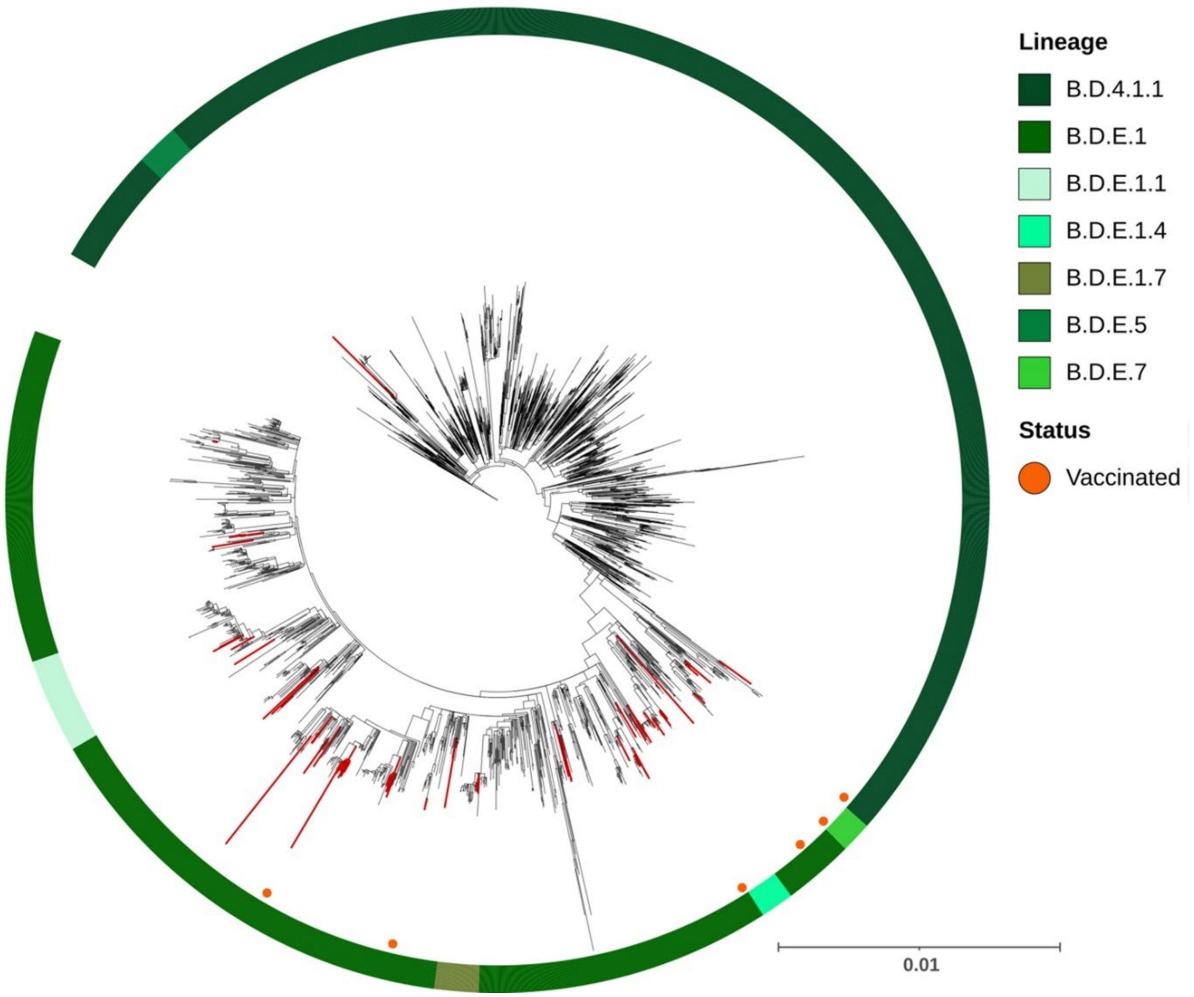

Appendix 1 Figure 2. Maximum-likelihood phylogenetic tree of human Respiratory Syncytial Virus subtype B (RSV-B) sequences. The analysis includes all 63 newly generated RSV-B genome sequences from this study together with 2,409 publicly available reference sequences. The phylogeny was inferred with IQ-TREE under the GTR+F+I+R7 nucleotide substitution model. Branch support was estimated using 1,000 ultrafast bootstrap replicates (UFBoot). The reference sequence dataset was genetically subsampled using a JC69-based proximity metric, retaining a maximum of 40 sequences per country per year. The red branches represent the sequences generated in this study, and the orange dots show the sequences obtained from vaccinated participants.

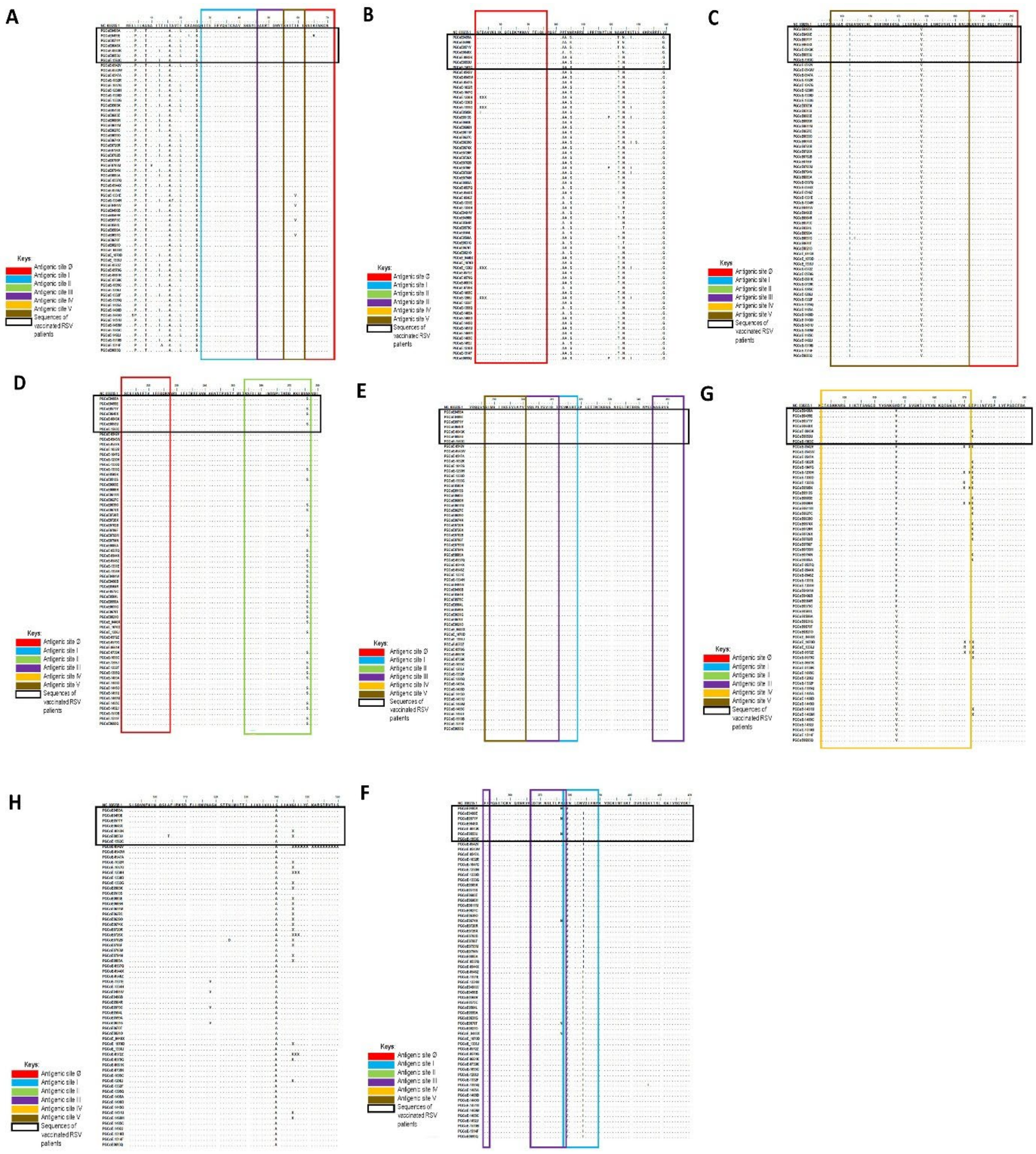

Appendix 1 Figure 3. (A-I) Amino-acid substitutions at the antigenic sites of the RSV-A F protein from vaccinated (black rectangle) and unvaccinated individuals. The 47 sequences generated in this study (labeled "PGCoE") were aligned to the RSV-A reference sequence NC_038235.1. The six antigenic sites are indicated by different colors: site Ø (red), site I (blue), site II (green), site III (yellow), site IV (brown), and site V (pink).

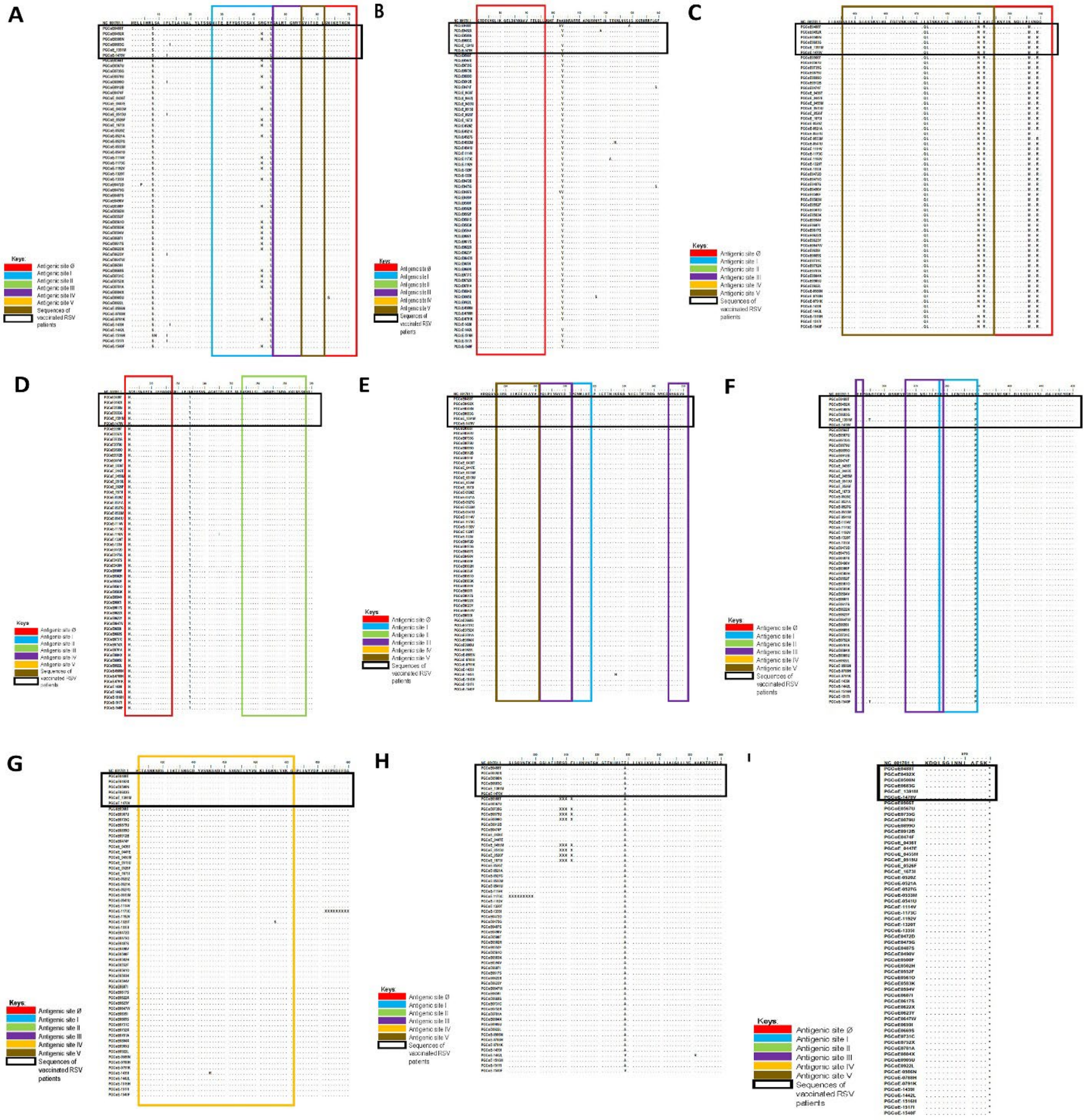

Appendix 1 Figure 4. (A-F) Amino-acid substitutions at the antigenic sites of the RSV-B F protein from vaccinated (black rectangle) and unvaccinated individuals. The 51 sequences generated in this study (labeled "PGCoE") were aligned to the RSV-B reference sequence NC_001781.1. The six antigenic sites are indicated by different colors: site Ø (red), site I (blue), site II (green), site III (yellow), site IV (brown), and site V (pink).
